# Age-Dependent Hepatic Involvement in Pediatric Epstein–Barr Virus Infection: Clinical Associations and Biochemical Recovery Patterns

**DOI:** 10.3390/jcm15062246

**Published:** 2026-03-16

**Authors:** Tuğba Gürsoy Koca, Dicle Şener Okur, Abdülkerim Elmas, Halil Kocamaz, Mustafa Akçam

**Affiliations:** 1 Department of Pediatrics, Division of Pediatric Gastroenterology, Hepatology and Nutrition, Pamukkale University Faculty of Medicine, Denizli 20070, Türkiye; tgkoca@gmail.com; 2 Department of Pediatrics, Division of Pediatric Infection, Pamukkale University Faculty of Medicine, Denizli 20070, Türkiye; diclesen71@yahoo.com; 3 Department of Pediatrics, Division of Pediatric Gastroenterology, Hepatology and Nutrition, Suleyman Demirel University Faculty of Medicine, Isparta 32100, Türkiye; makcam32@gmail.com; 4 Department of Pediatrics, Division of Pediatric Gastroenterology, Hepatology and Nutrition, Dicle University Faculty of Medicine, Diyarbakır 21280, Türkiye; drkocamaz@hotmail.com

**Keywords:** Epstein–Barr virus, hepatic involvement, pediatric cohort, logistic regression, biochemical recovery

## Abstract

**Background**: Epstein–Barr virus (EBV) infection is common in childhood and frequently accompanied by liver enzyme abnormalities. Although hepatic involvement is generally self-limited, pediatric data on predictors of hepatitis and biochemical recovery dynamics remain limited. This study aimed to evaluate the frequency and severity of hepatic involvement in children with primary EBV infection and identify clinical and laboratory features associated with hepatitis. **Methods**: This retrospective cohort study included children aged 0–18 years with serologically confirmed primary EBV infection at a tertiary center between January 2015 and November 2024. Patients with cytomegalovirus co-infection, hepatotropic viral infections, chronic liver disease, hepatotoxic drug exposure, or incomplete records were excluded. Demographic, clinical, and laboratory data were collected. Hepatic involvement was defined as ALT elevation above age-adjusted upper limits. Group comparisons utilized appropriate parametric and non-parametric tests, and logistic regression assessed predictors of hepatitis. **Results**: A total of 294 patients were included (median age: 6.1 years), of whom 48.6% were male. Hepatitis occurred in 59.2% of cases. Children with hepatitis were older than those without (median 6.7 vs. 5.3 years, *p* < 0.001). Logistic regression demonstrated that increasing age independently predicted hepatitis (odds ratio per year increase: 1.010; 95%CI 1.005–1.015; *p* < 0.001). Median time to ALT normalization was 20 days, and no patient developed acute liver failure. **Conclusions**: Hepatic involvement is common in pediatric EBV infection and is more frequent in older children. Despite significant biochemical abnormalities, all patients recovered fully with supportive care. Recognizing age-related risk and typical recovery patterns may reduce unnecessary investigations and guide appropriate management in pediatric EBV hepatitis.

## 1. Introduction

Epstein–Barr virus (EBV), a member of the Herpesviridae family, infects more than 90% of the global population and remains latent for life within B lymphocytes [1,2]. Primary infection typically occurs in childhood and is often asymptomatic; however, delayed exposure during adolescence or young adulthood may present as infectious mononucleosis (IM), characterized by fever, pharyngitis, and lymphadenopathy [3]. Hepatomegaly and mild to moderate elevations in liver aminotransferases are frequently observed during IM, reflecting hepatic involvement that is generally self-limited but occasionally severe [4]. The reported frequency of hepatic enzyme elevation in EBV infection varies between 80 and 90%, whereas clinically significant jaundice occurs in less than 10% of patients [3]. Hepatitis due to EBV is most often subclinical, resolving spontaneously within weeks, yet cholestatic hepatitis, acute acalculous cholecystitis, and even acute liver failure have been reported in rare pediatric cases [5,6]. The pathogenesis appears to be immune-mediated rather than cytopathic, as EBV has not been conclusively demonstrated within hepatocytes, in contrast to other herpesviruses such as cytomegalovirus [1]. Histologically, EBV hepatitis is characterized by sinusoidal lymphocytic infiltration with preserved lobular architecture, suggesting that liver injury results from cytotoxic T-cell responses and inflammatory cytokines rather than direct viral replication [7,8].

Age-related differences in hepatic response to EBV infection have been consistently observed. In large pediatric cohorts, older children and adolescents exhibit significantly higher alanine aminotransferase (ALT) and aspartate aminotransferase (AST) levels than preschoolers, with prolonged enzyme elevation and a higher proportion of atypical lymphocytes [9,10]. For instance, Wang et al. demonstrated that the incidence and magnitude of hepatic involvement in infectious mononucleosis increase with age, a finding corroborated by Son and Shin in Korean children and Arıkan et al. in a Turkish cohort [9,10,11]. A recent Polish study by Rutkowska and Pokorska-Śpiewak further reported that 46% of pediatric patients exhibited ALT levels exceeding five times the upper limit of normal, most commonly during the first week of illness, with transient cholestatic changes seen in approximately 10% [3].

Although hepatic dysfunction in EBV infection is usually benign, its clinical relevance lies in differential diagnosis and risk stratification. EBV hepatitis may mimic viral, autoimmune, or drug-induced liver injury, leading to unnecessary investigations or misclassification [4]. While several pediatric studies have described the frequency of transaminase elevation during infectious mononucleosis, detailed data regarding the determinants of hepatic involvement and the temporal dynamics of liver enzyme recovery remain limited, particularly in large pediatric cohorts. Furthermore, the clinical significance of age-related differences in hepatic involvement has not been sufficiently clarified, and evidence regarding whether age may serve as an independent predictor of EBV-associated hepatitis remains scarce.

Therefore, the present study aimed to evaluate the frequency and severity of hepatic involvement in children with primary EBV infection and to identify clinical factors associated with the development of hepatitis. In addition, we analyzed the temporal pattern of liver enzyme elevation and biochemical recovery, providing a comprehensive assessment of the natural course of EBV-related hepatitis in a large pediatric cohort. By clarifying the role of age as a predictor of hepatic involvement and describing the recovery dynamics of liver enzymes, our findings may help clinicians better interpret liver enzyme abnormalities in EBV infection and avoid unnecessary diagnostic investigations.

## 2. Methods

This retrospective observational study included pediatric patients aged 0–18 years who were diagnosed with primary Epstein–Barr virus (EBV) infection at the Department of Pediatrics, Pamukkale University Hospital, between January 2015 and November 2024. Patients were identified through the hospital’s electronic medical record system based on EBV serology results, and all consecutive eligible cases during the study period were screened for inclusion. Primary EBV infection was defined as the presence of immunoglobulin M (IgM) antibodies against EBV viral capsid antigen (VCA), detected by chemiluminescent immunoassay (CLIA) (LIAISON EBV IgM, DiaSorin S.p.A., Saluggia, Italy). Patients were considered to have acute or primary EBV infection if VCA IgM was positive and EBV nuclear antigen (EBNA) IgG was negative, based on a quantitative microplate ELISA (Euroimmun^®^, Lübeck, Germany). The onset of illness was defined as the first day on which symptoms such as fever, lymphadenopathy, sore throat, gastrointestinal complaints, or jaundice appeared. Hepatitis related to EBV infection was diagnosed when serum alanine aminotransferase (ALT) levels exceeded the age-adjusted upper limit of normal (ULN). The ULN for ALT in our laboratory was defined as 45 U/L according to the institutional reference range.

Patients were excluded from the study if they had cytomegalovirus (CMV) co-infection confirmed by positive CMV IgM serology, pre-existing liver disease or any chronic systemic disorder affecting hepatic function, positive serology for other hepatotropic viruses including hepatitis A, B, or C, a history of hepatotoxic drug use prior to disease onset, or incomplete medical records. Those who tested positive for EBNA IgG, indicating past infection, were also excluded. CMV IgM positivity without compatible clinical features or supportive laboratory findings was interpreted as serologic reactivity rather than confirmed acute CMV infection. Supportive laboratory findings for acute CMV infection included compatible clinical features together with elevated CMV IgM levels and, when available, confirmatory CMV PCR testing. In patients without compatible clinical findings, isolated CMV IgM positivity was interpreted as possible serologic cross-reactivity during acute EBV infection.

Demographic characteristics, clinical features, and laboratory data—including ALT, aspartate aminotransferase (AST), gamma-glutamyl transferase (GGT), total and direct bilirubin, complete blood count (CBC), and C-reactive protein (CRP)—were retrieved from the hospital’s electronic medical records. For the laboratory comparisons, the initial laboratory values obtained at hospital admission were used to ensure that only one measurement per patient was included in the analysis. Abdominal ultrasonography findings and treatment modalities administered during follow-up were also reviewed. For each patient, peak liver enzyme levels and the time to normalization were recorded. Patients with missing key data or alternative diagnoses were excluded from the final analysis. The severity of hepatic involvement was classified according to the degree of ALT elevation relative to the ULN: mild (1–3× ULN), moderate (3–5× ULN), and severe (>5× ULN). Because of the retrospective design, the study relied on previously recorded clinical and laboratory data; therefore, the possibility of selection and information bias cannot be completely excluded. Children with mild EBV infection who did not seek medical care may therefore not have been captured in this cohort.

Laboratory follow-up was performed according to routine clinical practice rather than a predefined protocol. Patients with abnormal liver enzymes were generally monitored with repeat laboratory testing until normalization or clear downward trends were observed. As this was a retrospective study, the timing and number of follow-up measurements varied between patients. Only patients with available follow-up laboratory data were included in the analyses evaluating time to peak levels and time to normalization.

Statistical analyses were performed using IBM SPSS Statistics for Windows, Version 27.0 (IBM Corp., Armonk, NY, USA). Continuous variables were tested for normality using the Shapiro–Wilk test. Data were expressed as mean ± standard deviation (SD) for normally distributed variables and as median (interquartile range, IQR) for non-normally distributed variables, whereas categorical variables were presented as frequencies and percentages. Homogeneity of variances was assessed using Levene’s test. Welch’s *t*-test was applied when unequal variances were detected. Comparisons between groups (hepatitis vs. non-hepatitis) were performed using Student’s *t*-test or Mann–Whitney U test for continuous variables and the chi-square or Fisher’s exact test for categorical variables, as appropriate. Correlations between liver enzyme levels and other laboratory or clinical parameters were analyzed using Spearman’s rank correlation coefficient. When multiple group comparisons were performed, post hoc pairwise analyses were conducted with Bonferroni correction to control for type I error. Multivariable logistic regression analysis was performed to identify independent predictors of EBV-related hepatitis. Multivariable logistic regression analysis was performed to identify independent predictors of EBV-related hepatitis. Age and sex were included as baseline demographic variables that could potentially influence susceptibility to hepatic involvement. Clinical manifestations, comorbidity status and laboratory parameters were not included in the regression model because these variables represent disease expression or severity after the onset of infection rather than baseline predictors. Prior to model construction, multicollinearity between independent variables was assessed using variance inflation factors (VIF), and no significant multicollinearity was detected. Results were expressed as odds ratios (OR) with 95% confidence intervals (CI). A two-tailed *p* value < 0.05 was considered statistically significant.

Ethical approval for the study was obtained from the Pamukkale University Faculty of Medicine Ethics Committee (Date: 26 November 2024, approval no: 20) and the study was conducted in accordance with the principles of the Declaration of Helsinki.

## 3. Results

A total of 294 pediatric patients with serologically confirmed primary EBV infection were included in the analysis. The patients’ median age was 6.08 years (IQR 3.7–9.6), and 48.6% (*n* = 143) were male. Hepatic involvement, defined as elevated ALT above age-specific reference values, was observed in 174 patients (59.2%). Additionally, 10.9% had at least one comorbidity. The majority of patients resided in urban areas (61.9%). Prior to hospital admission, 56.1% of patients had used medications, including antibiotics (18.7%) and a combination of antibiotics and antipyretics (30.3%). The median baseline ALT and AST levels were 63.5 U/L (IQR 19.3–193.8) and 56.5 U/L (IQR 32.0–143.3), respectively. Median white blood cell count was 12.2 × 10^3^/µL (IQR 8.6–16.7), and median platelet count was 245 × 10^3^/µL (IQR 190–314). EBV infection occurred most frequently in the spring months, with the highest peak in April (13.6%). All patients had serologically confirmed acute EBV infection, and those with alternative etiologies or incomplete records were excluded (Table 1).

Regarding clinical presentation, sore throat was more frequent among children who developed hepatitis compared with those without hepatitis (78.2% vs. 60.8%, *p* = 0.002). No statistically significant differences were observed between groups in terms of fever, rash, respiratory symptoms, or myalgia. Jaundice (5.8% vs. 0%, *p* = 0.006) and nausea/vomiting (23.0% vs. 10.8%, *p* = 0.007) were significantly more common in the hepatitis group. In addition, tonsillitis (78.1% vs. 55.8%, *p* < 0.001) and tonsillar exudate (52.2% vs. 36.6%, *p* = 0.008) were more frequent in patients with hepatitis. Hepatomegaly was also more common in children with hepatitis (29.8% vs. 15.0%, *p* = 0.003). CMV IgM seropositivity in the absence of clinical or laboratory findings consistent with acute CMV infection was observed more frequently in the hepatitis group compared with those without hepatitis (53.4% vs. 26.6%, *p* < 0.001). No significant differences were observed in lymphadenopathy, palpebral edema, splenomegaly, or hospitalization rates (Table 2).

When patients were stratified according to hepatic involvement, those with hepatitis were significantly older than those without hepatitis (median 6.7 years vs. 5.3 years, *p* = 0.0003). Children with hepatitis showed significantly higher AST levels and other markers of hepatic involvement compared with those without hepatitis. Laboratory comparisons between the two groups are summarized in Table 3. Within the hepatitis group, 44.8% of patients had mild hepatitis, 31.0% moderate hepatitis, and 24.2% severe hepatitis. Patients with hepatitis had higher lymphocyte counts and lower neutrophil and platelet counts compared to those without hepatitis. No significant differences were observed in hemoglobin, albumin, CRP, creatine kinase, or ESR levels between groups (Table 3).

**Figure 1:**
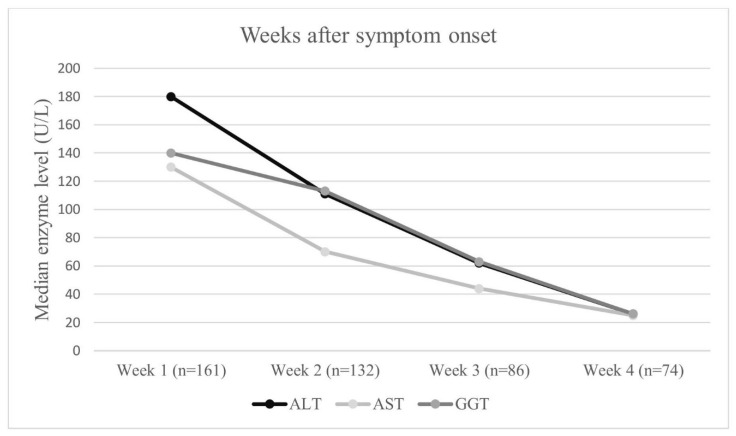
Temporal changes in median ALT, AST, and GGT levels during the first four weeks after symptom onset in children with EBV-associated hepatitis. The number of patients contributing data at each time point is shown on the x-axis. Abbreviations: ALT, alanine aminotransferase; AST, aspartate aminotransferase; GGT, gamma-glutamyl transferase.

ALT values normalized within a median of 20 days (IQR 14–30), while AST normalization required a mean of 19.5 days (IQR 10–28). GGT normalization occurred at a mean of 19.2 days. Total and direct bilirubin levels normalized at a median of 21 days (IQR 7–30). The mean time to peak transaminase elevation was 6.5 days after symptom onset, and all patients achieved full biochemical resolution within 60 days (Table 4). Temporal changes in liver enzyme levels during the first four weeks of illness are illustrated in Figure 1.

**Figure 2:**
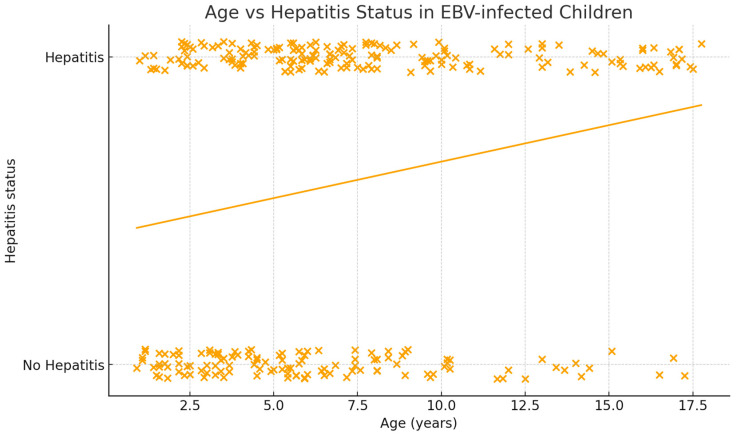
Age-related distribution of EBV-associated hepatitis in the study cohort. Each “x” represents an individual patient, plotted according to age and the presence of hepatitis. The *y*-axis represents hepatitis status (0 = no hepatitis, 1 = hepatitis). The line represents the fitted logistic regression trend illustrating the increasing likelihood of hepatitis with age.

In multivariable logistic regression analysis, increasing age was independently associated with the development of EBV-related hepatitis (odds ratio per year increase: 1.010; 95% confidence interval: 1.005–1.015; *p* < 0.001). Sex was not a significant predictor of hepatic involvement (Figure 2). When patients were stratified by age groups (<5 years, 5–10 years, and >10 years), the prevalence of hepatitis increased progressively with age. Hepatitis was observed in 45.7% of children younger than 5 years, 62.3% of those aged 5–10 years, and 74.6% of patients older than 10 years, demonstrating a clear age-related increase in hepatic involvement (*p* < 0.001) (Table 3).

## 4. Discussion

In this retrospective cohort of 294 children with primary EBV infection, hepatic involvement was identified in 59% of patients, confirming that liver enzyme elevation is a common manifestation in pediatric EBV disease. Children who developed hepatitis were significantly older than those without hepatic involvement, and age was independently associated with hepatitis, with each additional year increasing the odds by approximately 1%. Age-stratified analysis further demonstrated a progressive increase in the frequency of hepatitis across age groups, with the highest prevalence observed in children older than 10 years. This finding supports the regression analysis and suggests that hepatic involvement becomes more common with increasing age in pediatric EBV infection. Similar age-related differences in hepatic involvement have been reported in recent pediatric cohorts with infectious mononucleosis, in which older children were more likely to exhibit transaminase elevations and more pronounced hepatic involvement [12]. Clinical features such as sore throat, tonsillitis, and tonsillar exudate were more frequently observed in patients with hepatitis, while hepatomegaly and cholestatic markers, including GGT and bilirubin, were also elevated. Importantly, no cases of acute liver failure occurred, and biochemical abnormalities resolved within a median of 3–4 weeks, indicating a benign and self-limited course.

Our findings are consistent with previous pediatric studies demonstrating that hepatic involvement is common in EBV infection. In a Korean cohort, transaminase elevation was observed in more than half of hospitalized children with EBV infection, similar to the prevalence in our study. Another study reported hepatomegaly in approximately one-third of pediatric cases and noted that adolescents more frequently showed hepatic involvement compared with younger children [10]. These immune-mediated mechanisms may partly explain the age-related differences observed in our cohort. Several studies have suggested that stronger cell-mediated immune responses in older children and adolescents may contribute to a higher likelihood of hepatic involvement during primary EBV infection [1,4]. Previous reports also indicate that EBV-related hepatitis generally follows a benign course, with spontaneous resolution of biochemical abnormalities, which is consistent with the recovery pattern observed in our cohort [3,13,14].

The mechanism of EBV-associated hepatitis is believed to be predominantly immune-mediated rather than due to direct viral cytotoxicity. EBV infection triggers a robust cytotoxic T-cell response against infected B lymphocytes, leading to periportal and lobular inflammatory injury in the liver [2,4]. Increased hepatic involvement in older children may be explained by a more intense T-cell-mediated immune reaction and higher cytokine activity with advancing age, as suggested in prior pediatric studies [1,10]. Elevated lymphocyte counts and atypical lymphocytosis, observed in our cohort, are consistent with this immune-driven process, in line with reports demonstrating that activated CD8+ T cells and inflammatory mediators such as IL-2, IFN-γ, and TNF-α contribute to hepatocellular injury. Age-related differences in immune response may partly explain this observation. Younger children typically exhibit a less intense cytotoxic T-cell response to EBV-infected B lymphocytes, which may result in milder systemic and hepatic manifestations. In contrast, older children and adolescents tend to develop a more pronounced cell-mediated immune response, characterized by increased activation of CD8+ T lymphocytes and cytokine release. This heightened immune response may contribute to hepatocellular injury and, consequently, higher rates of biochemical hepatitis in older patients [15,16,17]. Recent pediatric studies have further suggested that heightened immune activation, reflected by increased cytokine responses during acute EBV infection, may be associated with a higher risk of hepatological complications [18].

From a clinical perspective, EBV-associated hepatitis in children typically follows a benign and self-limited course. In our cohort, no cases of acute liver failure occurred, and all biochemical abnormalities resolved spontaneously within a median of 3–4 weeks. These findings align with previous pediatric series reporting that although transaminase elevation is common during EBV infection, hepatic dysfunction rarely progresses to severe liver injury or liver failure [3,13,19]. Supportive management remains the mainstay of treatment, and routine use of antivirals or corticosteroids is not recommended in the absence of complications. Consistent with current evidence, early recognition of hepatic involvement may help prevent unnecessary investigation and interventions, particularly in children presenting with fever, tonsillitis, and elevated liver enzymes. An additional notable finding was the higher frequency of CMV IgM seropositivity among children with EBV-related hepatitis. This observation likely reflects serologic cross-reactivity during acute EBV infection rather than true CMV co-infection, as none of these patients exhibited clinical features suggestive of CMV disease. Previous studies have demonstrated that polyclonal B-cell activation and heterophile antibody production during EBV infection may result in transient CMV IgM reactivity, particularly in patients with more pronounced immune activation [20,21].

This study has several notable strengths. First, it represents one of the largest single-center pediatric cohorts evaluating hepatic involvement in primary EBV infection, providing a robust sample size for analysis. Second, all cases were serologically confirmed using VCA IgM and EBNA-IgG testing, ensuring accurate identification of true primary infections. Third, detailed laboratory follow-up allowed us to characterize the temporal dynamics of liver enzyme elevation and recovery, offering valuable insight into the natural course of EBV-related hepatitis. Furthermore, we identified age as an independent predictor of hepatic involvement through logistic regression analysis, contributing novel evidence to the emerging understanding that immune-mediated liver injury may be more pronounced in older children. Taken together, these strengths enhance the clinical relevance and reliability of our findings. From a clinical perspective, our findings suggest that liver enzyme monitoring may be particularly relevant in older children with primary EBV infection. Although EBV-associated hepatitis is usually self-limited, awareness of age-related differences in hepatic involvement may help clinicians anticipate the clinical course and plan appropriate follow-up.

This study has several limitations. First, its retrospective design may introduce potential selection and information bias. Although EBV infection was considered the primary cause of transaminase elevation in this cohort, the possibility of underlying metabolic-associated steatotic liver disease (MASLD) contributing to mild ALT elevations cannot be completely excluded in some patients. Second, although liver enzyme dynamics were carefully evaluated, detailed information regarding prior medication use, particularly antibiotics and antipyretic drugs frequently administered during febrile illnesses, was not consistently available in all patients. Such medications may potentially influence liver function biomarkers and could have contributed to variations in transaminase levels. Third, the study was conducted at a single tertiary care center, which may limit the generalizability of the findings to other populations and healthcare settings. Future multicenter prospective studies may help to better clarify the clinical spectrum and determinants of EBV-associated hepatitis in children.

In conclusion, hepatic involvement is common in pediatric patients with primary EBV infection and is more frequently observed in older children. Although transaminase elevations and cholestatic abnormalities were prevalent, all cases resolved spontaneously with supportive care, and no instances of acute liver failure occurred. Age was identified as an independent predictor of hepatitis, and biochemical recovery typically occurred within three to four weeks. Recognition of hepatic involvement in EBV infection may prevent unnecessary diagnostic testing and intervention, and our findings support a conservative management approach in otherwise stable pediatric patients.

## Figures and Tables

**Table 1:** Baseline demographic and clinical characteristics of children with primary Epstein–Barr virus infection.

Variables	Values
Total patients, *n*	294
Age, years, median (IQR)	6.08 (3.7–9.7)
Sex, male (%)	143 (48.6)
Hepatitis, *n* (%)	174 (59.2)
Comorbidity, *n* (%)	32 (10.9)
Residence; urban, *n* (%)	182 (61.9)
Medication use before hospital admission, n (%)	
None	129 (43.9)
Antibiotic	55 (18.7)
Antipyretic	20 (6.8)
Antibiotic and antipyretic	89 (30.3)
Other	1 (0.3)
ALT (U/L), median (IQR) (min–max)	63.5 (19.3–193.8) (6–1758)
AST (U/L), median (IQR) (min–max)	56.5 (32.0–143.3) (11–1735)
WBC (×10^3^/µL), median (IQR) (min–max)	12.2 (8.6–16.7) (1.2–54.4)
PLT (×10^3^/µL), median (IQR) (min–max)	245 (190–314) (10–640)

Variables with normal distribution are expressed as mean ± SD, while non-normally distributed variables are expressed as median (IQR). Normality was assessed using the Shapiro–Wilk test. Categorical variables are expressed as *n* (%). ALT: alanine aminotransferase; AST: aspartate aminotransferase; WBC: white blood cell count; PLT: platelet count.

**Table 2:** Clinical symptoms and physical examination findings in children with and without EBV-related hepatitis.

Symptoms	Hepatitis (+) *n* = 174	Hepatitis (−) *n* = 120	*p*
Fever, *n* (%)	125 (71.7)	76 (63.3)	0.17
Rash, *n* (%)	26 (15.0)	24 (20.0)	0.34
Sore throat, *n* (%)	136 (78.2)	73 (60.8)	**0.002**
Respiratory symptoms, *n* (%)	27 (15.7)	14 (11.7)	0.42
Myalgia, *n* (%)	31 (17.8)	12 (10.1)	0.09
Headache, *n* (%)	10 (5.7)	5 (4.1)	0.54
Jaundice, *n* (%)	10 (5.8)	0 (0)	0.006 *
Abdominal pain, *n* (%)	27 (15.5)	11 (9.1)	0.11
Nausea/Vomiting, *n* (%)	40 (22.9)	13 (10.8)	**0.007**
Diarrhea, *n* (%)	11 (6.3)	13 (10.8)	0.16
Duration of fever; days, median (IQR)	3 (1.2–5)	2 (0–4)	0.08
Duration of symptoms; days, mean (min–max)	6.4 (0–21)	7.48 (0–60)	0.27 **
Hospitalization, *n* (%)	63 (36.2)	33 (27.5)	0.11
Length of hospital stay; mean (min–max)	2.61 (0–15)	2.08 (0–17)	0.29 ^¥^
Tonsillitis, *n* (%)	136 (78.1)	67 (55.8)	**<0.001**
Tonsillar exudate, *n* (%)	91 (52.2)	44 (36.6)	**0.008**
Lymphadenopathy, *n* (%)	130 (74.7)	90 (75.0)	0.97
Palpebral edema, *n* (%)	24 (13.7)	8 (6.6)	0.052
Hepatomegaly, *n* (%)	52 (29.8)	18 (15.0)	**0.003**
Splenomegaly, *n* (%)	55 (31.6)	26 (21.6)	0.061
CMV IgM seropositivity without clinical CMV infection, *n* (%)	93 (53.4)	32 (26.6)	**<0.001**
Ultrasound-confirmed hepatomegaly, *n* (%)	32 (18.3)	10 (8.3)	0.055
Ultrasound-confirmed splenomegaly, *n* (%)	47 (27)	15 (12.5)	**0.015**
Gallbladder abnormalities on ultrasound, *n* (%)	8 (4.5)	0 (0)	0.05 *

Variables with normal distribution are expressed as mean ± SD, while non-normally distributed variables are expressed as median (IQR). Normality was assessed using the Shapiro–Wilk test. Bold values indicate statistically significant results (*p* < 0.05). Data are expressed as *n* (%). Comparisons were performed using the Chi-square test. Statistical significance was set at *p* < 0.05. * Fisher’s exact test; ** Welch *t*-test was used due to unequal variances (Levene *p* < 0.001); ^¥^ independent sample *t*-test.

**Table 3:** Laboratory parameters with and without EBV-related hepatitis.

Variables	Hepatitis (+) *n* = 174	Hepatitis (−) *n* = 120	*p*
Age (years), median (IQR) (min–max)	6.75 (4.3–10.5) (1–17.7)	5.33 (3.08–8.08) (1–17.2)	**<0.001**
Age groups, *n* (%)			**<0.001 ^§^**
<5 years (*n* = 105)	48 (45.7)	57 (54.3)	
5–10 years (*n* = 122)	76 (62.3)	46 (37.7)	
>10 years (*n* = 67)	50 (74.6)	17 (25.4)	
ALT (U/L), mean ± SD, (min–max)	202.5 ± 190.1 (46–1758)	19.7 ± 10.94 (6–45)	<0.001 *
AST (U/L), median (IQR) (min–max)	120 (65.5–199.25) (47–1735)	29.5 (23.25–37) (11–44)	**<0.001**
WBC (×10^3^/µL), median (IQR) (min–max)	13.2 (9.3–17.1) (1.2–43.3)	10.5 (7.5–16) (1.6–54.4)	**0.008**
Neutrophils (×10^3^/µL), median (IQR) (min–max)	2.88 (2.05–4.11) (0.9–31.0)	3.85 (2.57–5.99) (0.2–26.7)	**<0.001**
Lymphocytes (×10^3^/µL), median (IQR) (min–max)	8.64 (5.85–11.91) (1–22)	4.00 (2.42–8.33) (0.9–24.7)	**<0.001**
Monocytes (×10^3^/µL), median (IQR) (min–max)	0.58 (0.39–0.81) (0.4–3.2)	0.65 (0.45–1.00) (0.2–2.3)	**0.024**
Hemoglobin, (g/dL), median (IQR) (min–max)	12.3 (11.3–13.2) (8.6–22.1)	12.1 (11.3–12.8) (8.4–16)	0.147
Platelet (×10^3^/µL), median (IQR) (min–max)	228 (169–281) (4–640)	286 (224–339) (10–612)	**<0.001**
ALP (U/L), mean ± SD (min–max)	293.67 ± 147.20 (75–690)	179.08 ± 51.97 (67–349)	<0.001 *
GGT (U/L), median (IQR) (min–max)	88 (32–171) (11–479)	11 (9–17) (5–99)	**<0.001**
TBil (mg/dL), median (IQR) (min–max)	0.43 (0.31–0.87) (0.06–9.98)	0.23 (0.16–0.36) (0.1–0.8)	**<0.001**
DBil (mg/dL), median (IQR) (min–max)	0.24 (0.15–0.56) (0.04–9.6)	0.11 (0.09–0.17) (0.03–0.3)	**<0.001**
Albumin (g/L), median (IQR) (min–max)	41 (38–44) (25–52)	42 (40–44) (30–53)	0.22
LDH (U/L), median (IQR) (min–max)	491 (406.5–602.2) (214–1372)	310 (241–412) (151–960)	**<0.001**
CK (U/L), mean ± SD (min–max)	104.2 ± 225.6 (25–1339)	131.8 ± 163.3 (38–611)	0.067
CRP (mg/dL), median (IQR) (min–max)	2.9 (0.7–8.7) (0–222)	2.75 (0.4–11.5) (0.01–301)	0.72
ESR (mm/h), mean ± SD (min–max)	21.02 ± 16.6 (2–121)	23.8 ± 19.6 (2–79)	0.74

Variables with normal distribution are expressed as mean ± SD, while non-normally distributed variables are expressed as median (IQR). Normality was assessed using the Shapiro–Wilk test. Bold values indicate statistically significant results (*p* < 0.05). Comparisons between groups were performed using the Mann–Whitney U test. Statistical significance was set at *p* < 0.05. ^§^ Age group comparisons were performed using the chi-square test * Independent sample *t*-test. ALT: alanine aminotransferase; AST: aspartate aminotransferase; ALP: alkaline phosphatase; GGT: gamma-glutamyl transferase; LDH: lactate dehydrogenase; CK: creatine kinase; WBC: white blood cell count; ESR: erythrocyte sedimentation rate; CRP: C-reactive protein; TBil: total bilirubin; DBil: direct bilirubin.

**Table 4:** Time to peak liver enzymes and biochemical recovery in children with EBV-associated hepatitis.

Variables	Values
Time to peak transaminases, days (mean ± SD)	6.55 ± 7.38
Peak ALT (U/L), mean ± SD	200.6 ± 232.04
Time to AST normalization, days, median (IQR)	19.5 (10–28)
Time to ALT normalization, days, median (IQR)	20 (14–30)
Time to GGT normalization, days (mean ± SD)	19.2 ± 9.3
Time to total TBil normalization, days, median (IQR)	21 (7–30)
Time to DBil normalization, days, median (IQR)	21 (7–30)

Variables with normal distribution are expressed as mean ± SD, while non-normally distributed variables are expressed as median (IQR). Normality was assessed using the Shapiro–Wilk test. This table includes only patients with EBV-associated hepatitis. ALT: alanine aminotransferase; AST: aspartate aminotransferase; GGT: gamma-glutamyl transferase; TBil: total bilirubin; DBil: direct bilirubin.

## Data Availability

The data presented in this study are not publicly available due to privacy and ethical restrictions but are available from the corresponding author upon reasonable request.

## References

[1] Negro F. (2006). The paradox of Epstein-Barr virus-associated hepatitis. J. Hepatol..

[2] Leung A.K.C., Lam J.M., Barankin B. (2024). Infectious Mononucleosis: An Updated Review. Curr. Pediatr. Rev..

[3] Rutkowska M., Pokorska-Śpiewak M. (2023). Epstein Barr Virus Hepatitis-A Mild Clinical Symptom or a Threat?. Vaccines.

[4] Méndez-Sánchez N., Aguilar-Domínguez C., Chávez-Tapia N.C., Uribe M. (2005). Hepatic manifestations of Epstein-Barr viral infection. Ann. Hepatol..

[5] Shah J., Lingiah V., Pyrsopoulos N., Galan M. (2020). Acute liver injury due to severe Epstein-Barr virus infection. ACG Case Rep. J..

[6] Manappallil R.G., Mampilly N., Josphine B. (2019). Acute hepatitis due to infectious mononucleosis. BMJ Case Rep..

[7] Rigopoulou E.I., Smyk D.S., Matthews C.E., Billinis C., Burroughs A.K., Lenzi M., Bogdanos D.P. (2012). Epstein-barr virus as a trigger of autoimmune liver diseases. Adv. Virol..

[8] De Paschale M., Clerici P. (2012). Serological diagnosis of Epstein-Barr virus infection: Problems and solutions. World J. Virol..

[9] Wang Y., Li J., Ren Y.Y., Zhao H. (2013). The levels of liver enzymes and atypical lymphocytes are higher in youth patients with infectious mononucleosis than in preschool children. Clin. Mol. Hepatol..

[10] Son K.H., Shin M.Y. (2011). Clinical features of Epstein-Barr virus-associated infectious mononucleosis in hospitalized Korean children. Korean J. Pediatr..

[11] Arıkan K., Karadağ-Öncel E., Kara A., Cengiz A.B., Ceyhan M. (2023). Change of Laboratory Findings of Acute Epstein-Barr Virus Infection According to Age Groups. J. Pediatr. Infect..

[12] Zhang C., Cui S., Mao G., Li G. (2021). Clinical Characteristics and the Risk Factors of Hepatic Injury in 221 Children with Infectious Mononucleosis. Front. Pediatr..

[13] Zhang L., Zhou P., Meng Z., Pang C., Gong L., Zhang Q., Jia Q., Song K. (2018). Infectious mononucleosis and hepatic function. Exp. Ther. Med..

[14] Kofteridis D.P., Koulentaki M., Valachis A., Christofaki M., Mazokopakis E., Papazoglou G., Samonis G. (2011). Epstein Barr virus hepatitis. Eur. J. Intern. Med..

[15] Luzuriaga K., Sullivan J.L. (2010). Infectious mononucleosis. N. Engl. J. Med..

[16] Fugl A., Andersen C.L. (2019). Epstein-Barr virus and its association with disease—A review of relevance to general practice. BMC Fam. Pract..

[17] Hess R.D. (2004). Routine Epstein-Barr virus diagnostics from the laboratory perspective: Still challenging after 35 years. J. Clin. Microbiol..

[18] Moppert J., Domagalski K., Wrotek S., Pawłowska M. (2023). Are Selected Cytokines and Epstein-Barr Virus DNA Load Predictors of Hepatological Complications of Epstein-Barr Virus Infection in Children?. J. Clin. Med..

[19] Crum N.F. (2006). Epstein Barr virus hepatitis: Case series and review. South. Med. J..

[20] Landry M.L. (2016). Immunoglobulin M for Acute Infection: True or False?. Clin. Vaccine Immunol..

[21] Jhaveri T.A., Harris C., Sax P.E. (2022). IgM Positivity for Both EBV and CMV: A Clinical Conundrum. Open Forum Infect. Dis..

